# Exploring the evocative qualities of masks’ visual imagery and their associations with adversity and trauma

**DOI:** 10.3389/fpsyg.2024.1337927

**Published:** 2024-06-10

**Authors:** Asli Arslanbek, Bani Malhotra, Kristyn S. Stickley, Joanna Herres, Heather Spooner, Damon G. Lamb, Charles E. Levy, John B. Williamson, Girija Kaimal

**Affiliations:** ^1^Department of Creative Arts Therapies, Drexel University, Philadelphia, PA, United States; ^2^Department of Arts and Letters. The University of Tampa, Tampa, FL, United States; ^3^The College of New Jersey, Hamilton, NJ, United States; ^4^University of Florida, Gainesville, FL, United States; ^5^Henry M. Jackson Foundation for the Advancement of Military Medicine, Inc., Bethesda, MD, United States; ^6^Malcom Randall Veterans Affairs Medical Center, Gainesville, FL, United States

**Keywords:** trauma, imagery, masks, associations, emotional valence, emotional arousal, personal relevance, adversity

## Abstract

**Introduction:**

Studies suggest a relationship between the emotional evocativeness of visual imagery and viewer responses, however, there is limited understanding of these associations, especially as they relate to viewers’ personal experiences of adversities.

**Methods:**

In this exploratory study, we examined the relationship between the visual content of mask images and viewers’ responses. In an online survey 699 participants (of *n* = 1,010 total initial participants) rated 98 masks based on valence, arousal, and personal relevance and completed the Life Events Checklist. The masks included those created by service members (SMs) with traumatic brain injury (TBI), and post-traumatic stress disorder (PTSD), depicting physical, psychological, and moral injuries and matched neutral masks created by creative arts therapists and arts in health scholars.

**Findings:**

The findings indicated that responses to mask image content (traumatic versus neutral) were associated with viewers’ personal history of adversity and trauma. Specifically, images representing injury/trauma provoked stronger reactions on valence and arousal than neutral images. Moreover, participants with personal histories of trauma had heightened emotional responses to distressing imagery.

**Discussion:**

These findings have implications for art therapists as well as for clinical and general populations in that these results highlight the potential impact of distressing imagery particularly for individuals with personal histories of experiencing or witnessing traumatic events.

## 1 Introduction

Images of visual artworks are known to elicit empathetic reactions and evoke emotional responses (Kesner and Horáček, 2017). Freedberg and Gallese (2007) suggest that responses to artworks tend to be consistent across adult human viewers and consist of the initiation of embodied processes relating to actions, feelings, and bodily sensations. Discussing the impact of art and esthetic experiences, philosopher Langer (1966) posited that art serves the purpose of objectifying emotion to facilitate its understanding by both the artist and the viewer, essentially serving as education in emotional responsiveness because it enables individuals to develop empathy and gain an understanding of their own emotions and of those of others. Factors such as the arrangement of figures within the artwork, the composition of the artwork, contextual elements such as titles and accompanying texts, the art medium, the personality traits of the viewer, and cultural context all play a role in shaping the viewer’s affective reaction to the artwork (Leder et al., 2004; Kesner and Horáček, 2017).

Linking esthetic responses to neuroscience, Chatterjee and Vartanian (2014) proposed a triadic theory of artistic engagement that includes sensori-motor, emotional valuation and meaning making processes. Emerging neuroscience research on exploring the networks that support emotional response and personal relevance in the context of esthetic experiences (Vessel et al., 2013). Viewing an artwork engages our sensory- motor system: for example, dynamic brushstrokes can create a sense of movement and stimulating visual motion areas, or a landscape painting might evoke the parahippocampal gyrus area that is associated with recognizing places. Additionally, viewing objects that the viewer deems as beautiful, taps into the general reward circuitry, showing an emotional response toward imagery. Finally, the way we interpret or find meaning in art also influences our brain’s response: For instance, people shown an abstract artwork believed to be from a museum collection displayed more activity in the entorhinal cortex, a part of the brain involved in memory and association, compared to those who thought the artwork was created by a computer. This suggests that the significance we assign to art can affect how our brains react to it (Chatterjee and Vartanian, 2014).

Emotional valence is defined as an individual’s subjective response to a sensory stimulus, such that positive valence leads to approach-type behaviors and negative valence leads to avoidance-type behaviors (Pignatelli and Beyeler, 2019). The amygdala is implicated in both perception and behaviors related to the assignation of valence in responses. Emotional arousal refers to the intensity of the response to a stimuli (Citron et al., 2014). Together arousal and valence are associated with interoception and emotional awareness and important brain functions to help humans make adaptive behavioral responses. Esthetic experiences relate to the activation of sensorimotor areas, core emotional centers, and reward-related centers (Cinzia and Vittorio, 2009). The dorsolateral prefrontal cortex (DLPFC) plays a significant role in how beauty is perceived, as it is actively involved in our responses to esthetically pleasing images. This suggests that the evolution of the DLPFC may have a profound impact on our capacity for esthetic appreciation (Munar and Cela-Conde, 2021). Ardizzi et al. (2021) provided insights into the neural pathways activated when individuals view pain in artistic images. Their research highlighted the fact that activations in areas like the bilateral insular cortex, the posterior sector of the anterior cingulate cortex, and the anterior portion of the middle cingulate cortex, are indicative of empathic responses, which suggests that the level of activity in the empathic response regions directly correlates with the esthetic judgments formed by the participants. The research also underscored the intricate relationship between empathy for pain and the esthetic experience of artwork, suggesting that the way individuals perceive and evaluate pain in images is not based solely on its artistic context but is deeply rooted in innate empathic response mechanisms (Ardizzi et al., 2021). Research also shows that positive emotions, like joy and excitement, often activate regions such as the medial prefrontal cortex, which is associated with reward processing (Sabatinelli et al., 2007). On the other hand, emotions like fear and anger robustly engage the amygdala, a neural center vital for processing emotion (Adolphs, 2008). In the following section, we review the relevant literature on trauma, vicarious trauma and emotional responsiveness to imagery.

## 2 Trauma and vicarious trauma

Experiencing life events of extreme adversity and trauma can have impacts on emotional responsiveness (de Sousa et al., 2012). Traumatic experiences can result in a range of physical and psychological responses, such as intrusive flashbacks, dreams, or memories of the traumatic experience(s), avoidance of reminders of the trauma, emotional dysregulation, increased hypervigilance and activation when reminded of the trauma, and dissociation (American Psychiatric Association, 2013). Post-traumatic stress and TBI can often co-occur depending on the source of the injury (Vesterling et al., 2018). These responses can relate to interpersonal traumas such as physical or sexual assault, and non-interpersonal traumas encompass events that posed a severe threat to an individual’s life and well-being but are not directly caused by another person, such as natural disasters, accidents, or life-threatening illnesses (Hughesdon et al., 2021; Thomas et al., 2021). Comparatively, exposure to interpersonal traumas has a more detrimental impact on well-being than non-interpersonal traumas (Haldane and Nickerson, 2016). Individuals who have experienced interpersonal traumas exhibit higher levels of post-traumatic stress compared to those who have undergone non-interpersonal traumatic events (Thomas et al., 2021).

Relatedly, vicarious traumatization was coined as a term to describe the symptoms experienced by those who bear witness to the traumatic experiences of others (McCann and Pearlman, 1990). When healthcare providers empathetically attune themselves to their patient’s traumatic experiences, they become more susceptible to being vicariously traumatized, altering their perception of self, of others, and of the world around them (Pearlman and Mac Ian, 1995). Vicarious trauma has been found in professionals such as public service interpreters (Lai and Heydon, 2015), nurses (Isobel and Thomas, 2022), social workers (Méndez-Fernández et al., 2022), and law enforcement personnel within the criminal justice system (Burruss et al., 2018). Vicarious trauma responses can also occur in other segments of the population through media coverage of terror attacks, mass violence, or diseases (Ahern et al., 2004; Thompson et al., 2019; Liu and Liu, 2020). Vicarious trauma often goes unnoticed because individuals can still function effectively in their daily lives (Baird and Kracen, 2006). However, those who have witnessed traumatic events such as violence, accidents, or death may continue to carry the remnants of vicarious traumatization.

### 2.1 Assessing responses to visual imagery including masks

Human beings are inherently predisposed to react emotionally to the physical actions of others, including those depicted in artworks (Gallese, 2001, 2003; Freedberg and Gallese, 2007). This emotional engagement is not a mere passive observation; instead, it’s a visceral reaction that connects viewers to the actions they witness. Gallese (2001) argues that there may be a range of ‘mirror matching mechanisms’ present in our brains. When we observe someone performing an action, our motor system activates as if we were performing that same action, even if we do not physically replicate it. Moreover, neutral stimuli, such as masks devoid of distinct emotional expression, may elicit a less pronounced activation in these emotion-processing regions. However, the context in which these masks are presented can modify the viewer’s response. For instance, a neutral mask might evoke a different emotional reaction if viewed immediately following one that portrays intense fear or sorrow.

Masks have a long history and significance for humanity in various cultures around the world. They serve as an expressive tool for political thoughts, cultural identity, and the life cycle, while also representing peoples’ beliefs and emotional states. Artistic masks have been utilized in various cultures around the world as significant elements in protective rituals. These masks, often intricately designed, serve as more than mere esthetic objects: They embody a fusion of artistry and spirituality aimed at invoking protection or presenting personas in many parts of the world (Coldiron, 2005; Van Beek, 2018; Bertrand, 2020; Pan et al., 2020). In various cultures around the world these masks have a central role in the enactment and efficacy of fears and protective rituals. Given that masks are often used to scare away evil spirits in such mythological context (Speck, 1950), we can make connections between fearful neurological reactions and fear-inducing masks. Although it is clear that masks have a significant role in the history of humanity, a study of masks also begets the question: Is there a difference in humans’ neurological reactions to emotions displayed in masks versus emotions in real faces?

As a medium for exploring and expressing emotions, artistic masks possess the ability to convey a range of emotions, such as joy, tranquility, excitement and fear, anger, and anxiety. This inherent capability of masks ties directly to the human brain’s specialized mechanisms for processing emotionally charged visual stimuli. From a neurological standpoint, we are not aware of any study that directly compares human reactions to emotions in masks versus emotions in faces. Studies on emotional reactions to masks often focus on medical protective masks (Blazhenkova et al., 2022; Rinck et al., 2022). A study by Zhao et al. (2019) indicated that cartoon faces, compared to real faces, caused larger amplitudes in early neural processing stages, showcasing higher processing intensity and speed, but real faces garnered more attentional resources during later processing stages meaning that the degree of realism in facial representations could significantly impact neurological processing. Van den Stock et al. (2008) shed light on the importance of realistic emotional stimuli in understanding how the brain processes faces. These findings hinted at different neurological processing between real and non-real faces, which could extend to the comparison between real faces and artistic masks, suggesting that the realism and expressiveness of faces might influence the neurological and emotional reactions of individuals. Inferring from the sources discussed previously, if artistic masks are crafted to accurately reflect naturalistic emotional expressions, they may be activating the fusiform face area associated with recognition of faces and face-like images (Kanwisher et al., 1997). The FFA is located in the inferior temporal cortex and is typically larger in the right hemisphere of the brain. Masks being similar to faces in terms of shape and outlines of eyes, nose and mouth might elicit neurological responses in the fusiform face area (FFA) like those triggered by real faces. Conversely, if the emotional expressions in the masks are stylized or abstracted, the neurological responses might differ, potentially engaging different brain regions or processes. This suggestion emphasizes the potential role of artistic mastery and realism in crafting masks that can evoke neurological responses akin to those evoked by real faces.

With no direct comparison of neurological reactions to emotions portrayed in masks versus emotions portrayed in faces, studies on emotional stimulation and empathy within the context of general art viewing can also provide us with valuable information (Nadal, 2013; Potash et al., 2013; Vartanian and Skov, 2014; Boccia et al., 2016; Pearce et al., 2016). In a functional magnetic resonance imaging study (fMRI) on neural correlates of viewing delicate sadness represented by theatrical Japanese Noh masks when compared to neutral masks, Osaka et al. (2012) noted activation in the right amygdala likely due to the negative emotional processing thereby resembling what is typically seen with fear and disgust. Notably, when viewing faces that depict pain, as supported by the study of Ardizzi et al. (2021), specific neural pathways are speculated to be evoked in the brain as explained earlier. The pathways associated with empathy for pain include the bilateral insular cortex, which is pivotal for understanding others’ emotional states, the posterior sector of the anterior cingulate cortex (ACC) and the anterior portion of the middle cingulate cortex, regions deeply involved in processing distress and empathy. Additionally, the bilateral inferior frontal gyrus and the posterior cingulate cortex/precuneus, especially activated for non-artistic stimuli, play significant roles in this empathic response. These neural mechanisms underline how humans empathetically connect with and understand the emotional states of others, especially pain, even when presented artistically.

Specific to the PTSD, trauma-induced changes in the brain’s key regions, such as the amygdala, medial prefrontal cortex, and hippocampus, can influence the interpretation of visual cues and the associated emotional responses (Hendler et al., 2003; Shin et al., 2006). Hendler et al. (2001) suggest that traumatic experience may modulate brain activity at the level of the sensory cortex. Individuals with PTSD might exhibit heightened sensitivity or reactivity to traumatic visual stimuli (Bremner et al., 1999; Lanius et al., 2002). In another study, Mueller-Pfeiffer et al. (2013) showed that participants exposed to trauma with and without a diagnosis of PTSD showed differential responses visual stimuli. Those with a PTSD diagnosis rated images as more arousing and less pleasant than the control participants, who were exposed to trauma, but did not qualify for PTSD. Taken together, the neurological consequences of both TBI and PTSD can substantially modify an individual’s response to visual imagery, underscoring the brain’s vulnerability and adaptability.

### 2.2 Artistic masks and art therapy

In the profession of art therapy, mask-making has been recognized as a safe and effective medium that enables individuals to establish psychological distance for the purpose of self-expression (Jones et al., 2018; Kaimal et al., 2018; Maltz et al., 2020) and has been used to depict transformation and growth (Dunn-Snow and Joy-Smellie, 2000). Masks have been used in art therapy across different developmental stages and with a range of populations. Including youth (Feen-Calligan et al., 2020) and with women who experienced incarceration and bereavement (Ferszt et al., 2004). According to Wadeson (2000), creating and engaging with masks is unique in that “they allow us to become what we are not,” which may support clients in sharing their thoughts and feelings in new ways (p. 420).

The act of creating masks provides valuable therapeutic opportunities by externalizing internal conflicts, containment of overwhelming emotions, and engaging in self-exploration and self-integration (Walker et al., 2017). This practice has also demonstrated its efficacy in assisting military personnel in visualizing and expressing memories associated with combat, while simultaneously promoting self-efficacy and the normalization of emotions (Walker et al., 2016, 2017). Walker et al. (2017) highlighted how mask-making enabled service members (SMs) to visually represent their experiences of post-traumatic stress disorder (PTSD) and TBI in ways words alone could not capture. The physical representation of ideas portrayed in the masks has been associated with unspoken ideas of physical and psychological injuries as well as with moral injuries of guilt, loss and shame. These visual elements were also found to be associated with standardized measures of PTSD, depression and anxiety in service members with combat-related TBI (Kaimal et al., 2018). The SM masks used in this current study are for the public domain (Alexander, 2015a) and are a subset of those evaluated in the study by Walker et al. (2017) and Kaimal et al. (2018)’s studies. Therefore, the images evaluated by our participants were previously studied for their portrayal of graphic experiences associated with PTSD and TBI.

### 2.3 Rationale and hypotheses for the current study

Researchers have examined the phenomenon of affective responses to artwork. However, to the best of our knowledge, no previous studies have specifically explored an affective comparison between two types of face mask imagery as well as personal history of a range of traumatic experiences. The first type includes masks created by military service members with a history of post-traumatic stress disorder (PTSD) and traumatic brain injury (TBI) as part of their art therapy treatment. The second type consists of replicas of these masks, designed by a team of creative art therapists and arts and health research students to be content-neutral. Our study aims to investigate the emotional activation that results from viewing both types of masks in the general population of viewers.

Assessments of evocative impacts of viewing qualities of artistic works (for example depicting facial structures in masks) have not been explored. The goal of this present study was to investigate the influence of mask imagery created by SMs with PTSD and TBI, juxtaposed with comparatively matched neutral imagery, on the emotional responses of viewers. This approach becomes especially pertinent considering that individuals with traumatic or adverse life events might exhibit heightened or altered neural reactions to certain emotional stimuli as explained earlier. By focusing on the emotional responses elicited by these masks, this study aims to offer insights into how trauma and adversity influence perception and emotional reaction toward traumatic imagery in art, contributing to the broader field of neurological responses to artistic works. We are not aware of any studies to date that have assessed the responses to viewing visual imagery and personal experiences of adversity.

The hypotheses that were tested in this exploratory study were as follows:

1. Hypothesis 1: Masks created by service members (SM) will elicit a stronger emotional response (in terms of arousal, valence and personal relevance) among participants compared to neutral masks.
2. Hypothesis 2: The emotional response (in terms of arousal, valence and personal relevance) to the SM masks will be more pronounced for individuals with a reported personal history of trauma and adversity.

Findings from this study can help inform art therapists and researchers as well as anyone interacting with individuals who have had experiences of adversity and trauma on the potential behavioral reactions and responses to evocative imagery.

## 3 Methods

### 3.1 Study design

This study used a within-subject design to investigate the influence of content and formal elements in artistic masks on the emotional responses of viewers. Participants were invited to evaluate a collection of masks created by both civilians and military service members. The assessment of each mask image was based on three dimensions tested in IAPS: affective valence, arousal, and personal relevance. The dominance dimension in IAPS was changed to personal relevance for this study to understand the degree to which the image is relevant to the viewer. The viewers rated the mask images using an adapted version of the Self-Assessment Manikin (SAM) used in norming the International Affective Picture System (IAPS) (Lang et al., 2005).

Two sets of masks were presented to the public for evaluation through an online survey. One set was created by SM with a history of traumatic brain injury (TBI) and post-traumatic stress disorder (PTSD). These masks included content relevant to the experience of TBI and PTSD, representing subject matter derived from the artists’ personal experiences. SM masks were selected based on their intensity and were indicative of physical, moral, or emotional injuries. The second set of masks were created by a team of creative art therapists, and arts and health research students, mirroring the SM masks in color, shape and form without the traumatic content. The order of the images presented to the participants was randomized by the survey measure. At the end of the survey, the participants were asked to complete the Life Events Checklist (LEC), an assessment that measures the amount of significant change in one’s life over the past 12 months (Weathers et al., 2013). The inclusion of the Life Events Checklist aimed to explore the potential influence of recent life-changing events on participants’ responses to the imagery. The study received an approval from the Institutional Review Board of Drexel University (protocol # 2204009167A002).

### 3.2 Measured

The Life Events Checklist (LEC) is a self-report questionnaire used to determine the participant’s level of exposure to various potentially traumatic experiences (Gray et al., 2004). The LEC was originally created alongside the Clinician-Administered PTSD Scale for the *Diagnostic and Statistical Manual of Mental Disorders, 4th Edition* (DSM-IV) based on *DSM-IV* diagnostic criteria for Post-Traumatic Stress Disorder (PTSD) and has been updated to the LEC-5 with minor changes, based on traumatic experiences that often contribute to the formation of PTSD as outlined in the *DSM-V* (National Center for PTSD, 2023). The LEC-5 has 16 items representing various kinds of traumatic events (and one item that can include a traumatic experience not accounted for by the previous 16 items) (National Center for PTSD, 2023). For each item, the participant rates their level of exposure to the type of trauma using responses such as: “happened to me,” “witnessed it,” “learned about it,” “part of my job,” “not sure,” and “does not apply” (National Center for PTSD, 2018). The LEC appears “to be characterized by generally adequate psychometric properties” and is comparable to other measures of traumatic exposure [Clinician-Administered PTSD Scale for the *Diagnostic and Statistical Manual of Mental Disorders, 4th Edition*, the PTSD Checklist for DSM-5 (Military), and the Mood and Physical Symptoms Scale] when tested with both students and combat veterans (Gray et al., 2004, p. 336). However, when testing the psychometric properties of the LEC, the sample for studies conducted by Gray et al. (2004) demonstrated a lack of racial diversity, with the majority of participants identifying as white. There is limited data on the psychometric properties for the updated LEC-5. However, Pugach et al. (2020) studied the LEC-5’s test/retest reliability at intervals of 8 and 12 weeks, and found that the LEC-5’s reliability was most consistent pertaining to traumatic experiences that were “directly experienced” (such as “reports of sexual assault, physical assault, transportation accidents, natural disasters, and other sexual experiences”), rather than traumatic experiences that did not directly happen to participants (p. 248).

**Figure 1:**
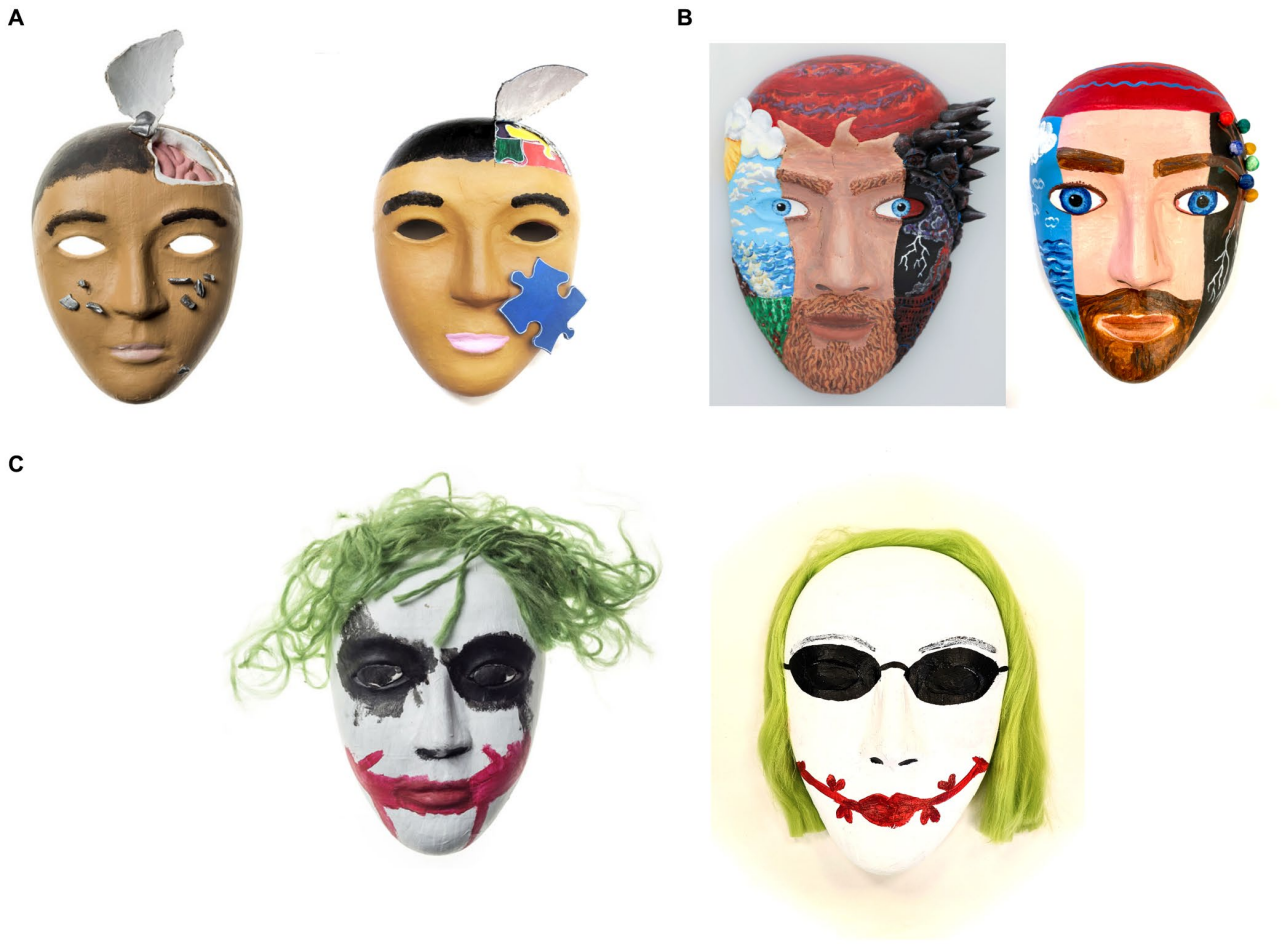
**(A)** Original mask created by a military member (left) and a neutral mask created by civilian art therapist (right). The mask on the left is cut around the head area with exposed pink material reminiscent of a brain with metal-like clay pieces attached on the cheeks (this mask has been previously published in Walker et al. (2017); Permission to reuse the picture obtained). The neutral mask on the right also has a cut on the left side of the head with colorful puzzle pieces with a blue single puzzle piece attached on the right cheek. **(B)** Original mask created by a military member (left) and a neutral mask created by civilian art therapist (right). The mask on the left is divided in two. One section depicts a scenery with blue and green colors, suggesting a landscape, while other section is colored in black with thunderstorms and spikes erupting from the temples (this mask has been previously published in Kaimal et al. (2018); Permission to reuse the picture obtained). The neutral mask on the right also is divided in two: one side depicts the sea and greenery while other side is colored in black. However, different than the SM mask, the neutral mask has colorful pompoms and the thunderstorm morphs into tree roots. **(C)** Original mask crated by a military member (left) and a neutral mask created by civilian art therapist (right). The mask on the left portrays the comic book character, Joker. It has smudged paint around the eyes, a mouth painted with broad red lines mimicking Joker’s smile, and its top is decorated with disheveled green felt imitating messy hair (this mask has been previously published in Walker et al. (2017); Permission to reuse the picture obtained). The mask on the right side features black sunglasses, a mouth marked by precise thin red lines with little hearts, and yarn arranged to look like neatly combed hair draping down from both sides.

The masks created by SMs with a history of TBI and PTSD were previously published in a research paper (Walker et al., 2017) and were publicly available as they were published online by National Geographic (Alexander, 2015b). We asked participants to rate each image in order to understand how the content and formal qualities evoked emotional responses in the viewer. The images were chosen from a publicly available source (Alexander, 2015b) and no identifiers were used (see Figures 1A–E for examples of modified matched neutral images). The masks were not analyzed for viewer responses to specific elements, rather the overall responses to the imagery were broadly categorized into SM masks (with explicit traumatic content) and neutral masks (with the traumatic content reimagined). Successful evocation of an emotional response with visual imagery may be useful in controlled studies of emotion. However, this research is particularly interested in the relationship of personal experience with traumatic content. The use of artistic content from SMs with real-life experiences of PTSD may be specifically useful in studies of individual differences in response to trauma relevant content.

Various scales have been developed to evaluate the formal emotional and expressive qualities of images. One such scale is the Self-Assessment Manikin (SAM) (Lang et al., 1997). SAM is a picture rating system that comprises of a set of three graphic scales that represent three dimensions of emotional experience: valence, arousal, and dominance. Valence represents the positivity or negativity of the emotional response, arousal indicates the intensity or activation level of the emotion, and dominance reflects the sense of control over the emotion. The Self-Assessment Manikin (SAM) was developed as a tool to measure emotional responses and subjective feelings in various research studies. It was created to provide a simple and standardized way for individuals to express their emotional experiences in response to different stimuli, such as images, videos, or events. It offers a non-verbal and culturally neutral means for individuals to convey their emotional experiences, making it suitable for cross-cultural studies and populations with limited verbal abilities, such as children or individuals with cognitive impairments. SAM was also used in evaluating a set of emotionally evocative imagery in the International Affective Picture System (IAPS) (Lang et al., 2005). The IAPS is a collection of standardized images that are used in fMRI studies to examine emotional responses and reactions in psychological research. Lang et al. (2005) designed IAPS to facilitate the investigation of how different visual stimuli evoke emotional reactions and how these reactions might vary across individuals. The IAPS comprises thousands of images that cover a broad range of emotional valence (pleasantness or unpleasantness) and arousal (intensity of emotional response) and dominance. These images can depict scenes, objects, animals, and people, and they are scaled along these three dimensions of valence, arousal, and dominance. Drawing a parallel to the mechanism of evaluating emotional response through imagery, artistic masks emerge as a potent medium for exploring and expressing a spectrum of emotions, further enriching the discourse on visual stimuli and emotional response.

**Figure 2:**
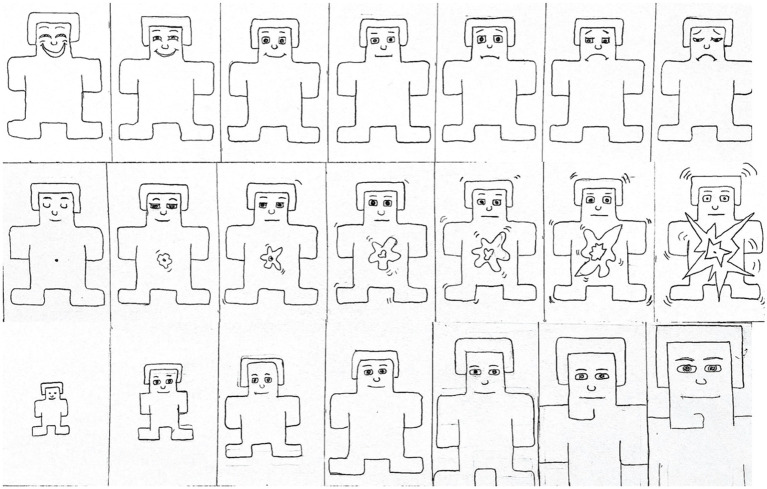
Adapted Self-Assessment Manikin for valence (top row), arousal (second row), and personal relevance (bottom row).

The SAM has been widely used as a standardized measure of emotional and cognitive properties of imagery. For this research, we used a modified 7-scale version of SAM, and asked each survey responder to evaluate the images. In the original IAPS normative approach, a dominance scale was used to represent the extent to which participants felt that they had control over their emotions However, for this research, we substituted “personal relevance” for “dominance,” in order to determine to what extent participants reported that the imagery was relevant to their experiences. We also modified the original SAM to include seven images instead of five because our goal was to provide participants with more nuanced levels of emotional expression through a diversified alternative to the original scale (Figure 2).

### 3.3 Recruitment

Survey participants were recruited through flyers on the departmental website, standard research outreach through listservs, and ResearchMatch, a web-based recruitment registry connecting volunteers with research studies (Harris et al., 2012). The survey participants’ inclusion criteria were proficiency in English and being over the age of 18, and exclusion criteria were being active-duty military members and or having active-duty military members in their immediate families. In the study flyer, we explained the study, and highlighted the importance of understanding the minimal risk involved. The risk of harm was minimal as the only task involved in this research was completing an online survey in which participants were asked to evaluate artworks created by SMs and civilians and to complete the Life Events Checklist. Participants were, however, given the disclaimer that some images could be disturbing and that they could choose to leave the survey at any time. It was up to the individual to decide to proceed by clicking on the link and completing the online survey, and the participant could choose to leave the survey at any time. The team sent information from the study flyer to up to 1,500 potential participants per day from the ResearchMatch database. Of those, volunteers who chose to indicate that they were interested in the study were then sent the link to our survey. In the end, the flyer was sent to 40,497 potential participants through Research Match and *n* = 1,010 consented to participate.

### 3.4 Procedures

Through an online survey, adult participants aged 18 years or older and proficient in English were presented with a total of 98 masks. Participants were requested to rate each mask in terms of its evocative qualities, specifically valence, arousal, and personal relevance. The survey participants clicked on the link provided that led to an online survey that included 98 images of combined SM and neutral masks. The first page of the survey required the participants to complete an informed consent form that had been approved by Drexel University’s institutional review board. On average, participants were expected to complete the online survey within 30 min; however, the survey took on average 60 min to complete in full. Data were gathered using an online survey platform called Qualtrics (Qualtrics, Provo, UT, United States). The participants were anonymous, and the research did not involve disclosure of identifying information.

### 3.5 Data analysis

We analyzed the survey data using SPSS version 27 (IBM-SPSS, Armonk, NY, United States). Descriptive statistics and model diagnostics were performed to confirm that assumptions were met prior to analyses. For hypothesis 1, paired samples *t*-tests compared the mean valence, arousal, and personal relevance ratings for the trauma vs. neutral masks to test whether the trauma masks evoked a stronger response compared with the neutral masks. A series of mixed model factorial analyses of variance (ANOVAs) using Type III SS tested hypothesis 2 to determine the effects of the mask type (trauma vs. neutral) and trauma context (either experienced, witnessed, learned about, or experienced as part of the job) on valence, arousal, and personal relevance ratings. A second series of mixed ANOVAs using Type III SS examined the interactions between the type of traumatic event the participants experienced and the type of mask on the three outcome variables (valence, arousal, and personal relevance).

## 4 Results

The obtained results indicated differences in how participants rated the SM masks versus the neutral masks, and correlations between the participants’ own exposure to trauma and how the masks were rated. Participation in the survey dropped from 1,101 participants responding to mask questions in the first half of the survey to 699 participants (63.5%) progressing through the Life Events Checklist at the end. There were no differences between those who completed the full survey and those who only completed the first half in overall valence [*M_Diff_* = −0.02, *t* (523.24) = 1.08, *p* = 0.279] and arousal ratings [*M_Diff_* = 0.06, *t* (1040) = −0.23, *p* = 0.818]. Those who completed the full survey rated the masks lower in personal relevance than those who did not [*M_Diff_* = 0.18, *t* (613.62) = 2.31, *p* < 0.05], suggesting a systematic bias to missingness. Overall ratings for those who progressed to the end of the survey showed that on average, participants found both set of masks collectively to be only slightly more unpleasant than pleasant (*M* = 4.32, *SD* = 0.70), moderately arousing (*M* = 3.75, *SD* = 1.12), and relatively low in relevance (*M* = 2.81, *SD* = 1.12). Subsequent analyses were conducted only on participants with complete survey data, though we verified that the patterns of results were the same when those who did not complete the full survey were included in analyses. The two hypotheses and related reports from the analyses are included below:

### 4.1 Hypothesis 1

SM masks will elicit a stronger emotional response (in terms of arousal, valence and personal relevance) than neutral masks.

Paired samples *t*-tests showed that participants rated the SM masks as having more negative valence (*M* = 4.93, *SD* = 0.71) than the neutral masks (*M* = 3.69, *SD* = 0.70), *t* (697) = −42.94, *d* = 0.88, *p* < 0.01. Arousal ratings were also higher for the SM masks (*M* = 4.12, *SD* = 1.13) compared to the neutral masks (*M* = 3.39, *SD* = 1.09), *t* (697) = −32.72, *d* = 0.71, *p* < 0.01. On average, participants indicated that the neutral masks resonated more on personal relevance (*M* = 2.82, *SD* = 1.09) than the SMa masks (*M* = 2.69, *SD* = 1.14), *t* (698) = 5.16, *d* = 0.80, *p* < 0.01. The effect sizes (*d)* ranged from 0.71 and 0.88, indicating medium-large to large effects.

### 4.2 Hypothesis 2

The emotional response (arousal, valence, and personal relevance) to the SM masks will be more pronounced for individuals with a reported personal history of trauma and adversity.

**Figure 3:**
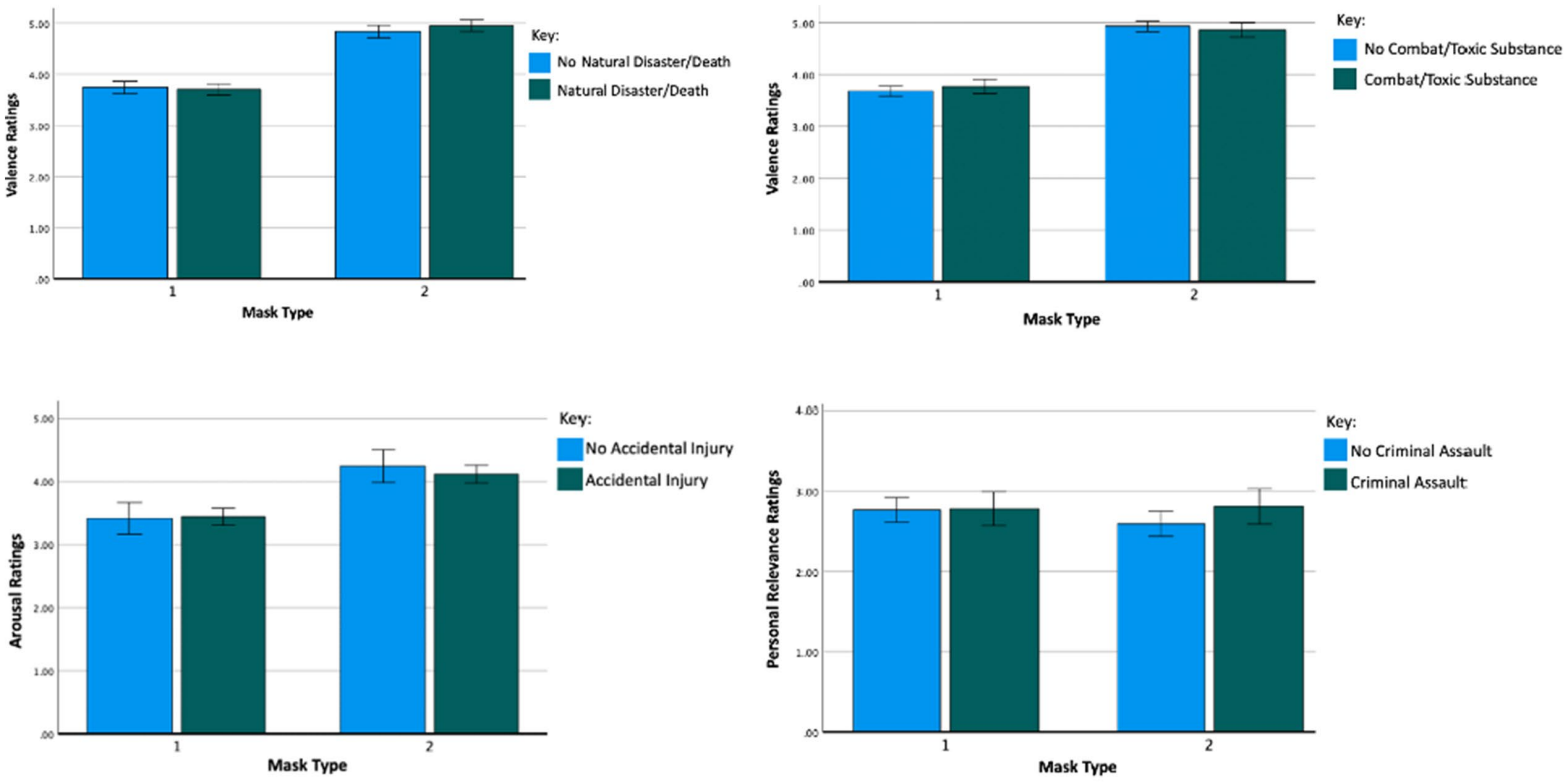
Interaction effect (mask type X trauma type) on valence, arousal, and personal relevance ratings with error bars representing the 95% C.I. Results demonstrate that there were no main effects of any of the trauma context variables and no interaction effects between mask type and any of the trauma context variables on valence, arousal, and personal relevance. This figure demonstrates the interaction effects.

**Figure 4:**
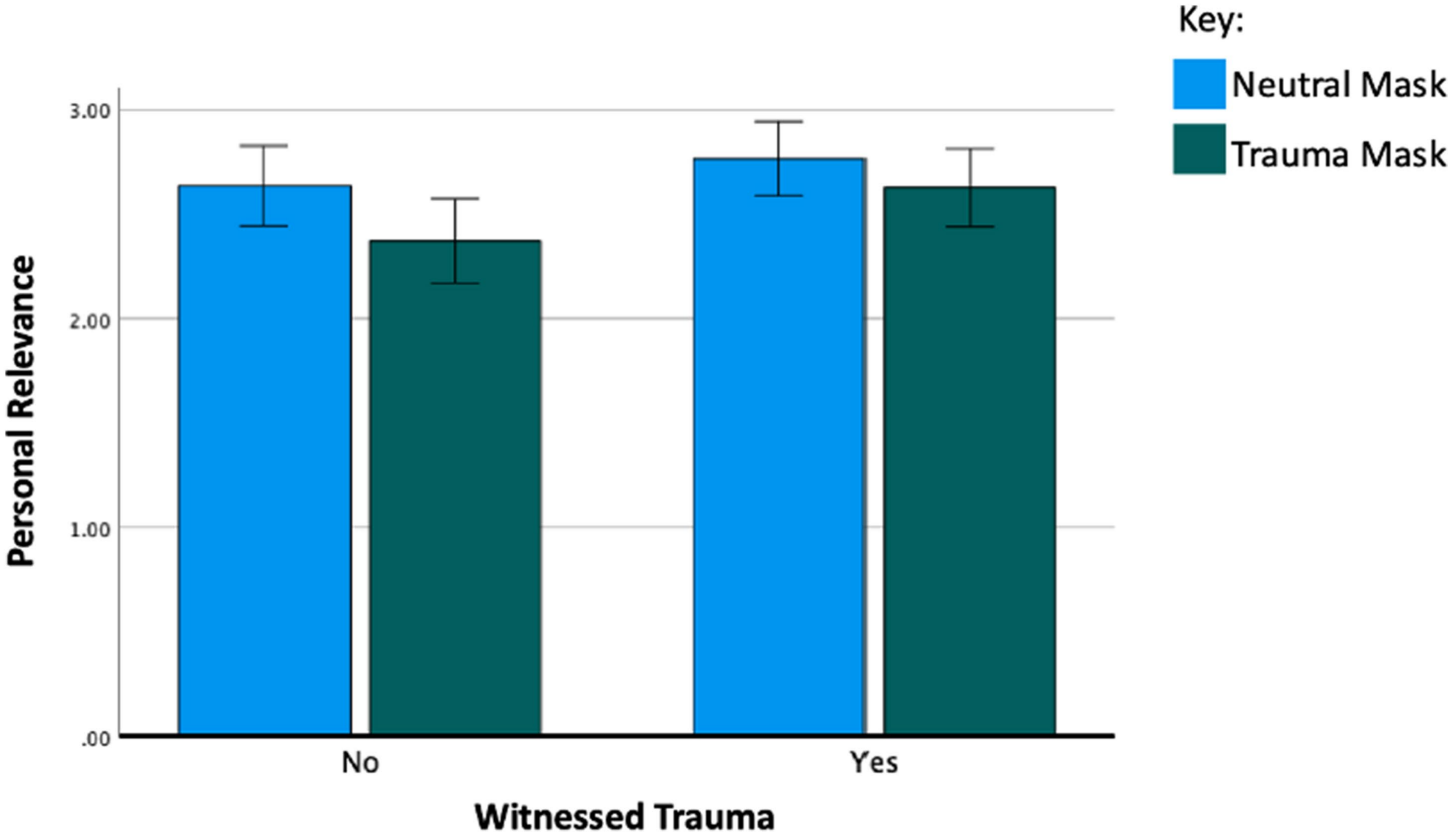
Interaction effect (mask type X witnessed trauma) on personal relevance ratings with error bars representing the 95% C.I. Results show a main effect of mask type on personal relevance (η*_p_*^2^ = 0.02), a main effect of witnessing trauma on personal relevance (η*_p_*^2^ = 0.01), and an interaction effect between witnessing trauma and mask type (η*_p_*^2^ = 0.01). This figure demonstrates the interaction effect. Participants who had not witnessed trauma rated the neutral masks more personally relevant. Participants who had witnessed trauma found both neutral masks and trauma masks equally personally relevant. Overall, participants who had witnessed trauma found both masks more relevant compared to those who had not witnessed trauma.

Table 1 shows the frequency with which the participants endorsed having experienced each of the different types of traumatic events. Table 2 shows analysis of variance results examining the effects of trauma context and Table 3 shows results examining effects of type of trauma. While there were significant main effects of mask type on both arousal (η*_p_*^2^ = 0.29) and valence (η*_p_*^2^ = 0.399), there were no main effects of any of the trauma context variables and no interaction effects between mask type and any of the trauma variables (Figure 3). Results for personal relevance showed a main effect of mask type (η*_p_*^2^ = 0.02), a main effect of witnessing trauma (η*_p_*^2^ = 0.01), and an interaction effect between witnessing trauma and mask type (η*_p_*^2^ = 0.01). Whereas participants who had *not* witnessed trauma rated the neutral masks more personally relevant than the SM masks, those who had witnessed trauma found both sets of masks to be equally personally relevant, overall rating both sets more relevant than those who had not witnessed trauma (Figure 4). Several interactions between type of traumatic event and mask type were significant (see Table 3; Figure 2). However, the effect sizes were small (η*_p_*^2^ = 0.001 to 0.008).

**Table 1:** Frequencies with which participants experienced the different types of traumatic events.

	Happened to me		Witnessed it		Learned about it		Part of my job	
Trauma type	f	%	f	%	f	%	f	%
Natural disaster (*n* = 695)	248	35.68	181	26.04	282	40.58	40	5.76
Fire or explosion (*n* = 689)	70	10.16	153	22.21	252	36.57	42	6.10
Transportation accident (*n* = 690)	384	55.65	232	33.62	254	36.81	60	8.70
Serious accident (*n* = 692)	101	14.60	125	18.06	228	32.95	77	11.13
Exposure to toxic substance (*n* = 688)	63	9.16	47	6.83	147	21.37	59	8.58
Physical assault (*n* = 694)	270	38.90	180	25.94	245	35.30	74	10.66
Assault with weapon (*n* = 687)	67	9.75	57	8.30	235	34.21	61	8.88
Sexual assault (*n* = 693)	216	31.17	37	5.34	292	42.14	75	10.82
Other unwanted sexual performance (*n* = 692)	335	48.41	81	11.71	249	35.98	64	9.25
Combat or exposure to war zone (*n* = 690)	16	2.32	30	4.35	231	33.48	38	5.51
Captivity (*n* = 688)	28	4.07	17	2.47	132	19.19	19	2.76
Life-threatening injury/illness (*n* = 693)	199	28.72	255	36.80	252	36.36	82	11.83
Severe human suffering (*n* = 691)	44	6.37	151	21.85	240	34.73	101	14.62
Sudden violent death (*n* = 687)	25	3.64	73	10.63	330	48.03	77	11.21
Sudden accidental death (*n* = 682)	23	3.37	101	14.81	298	43.70	66	9.68
Serious injury, harm, or death (*n* = 690)	21	3.04	26	3.77	58	8.41	21	3.04
Other stressful event (*n* = 689)	345	50.07	156	22.64	172	24.96	92	13.35
Any trauma (*n* = 699)	634	90.7	519	74.2	589	84.3	236	33.8

**Table 2:** Results from analysis of variances examining differences in arousal, valence, and personal relevance ratings by mask type, trauma history across contexts, and the interactions between mask type and trauma history.

	Arousal		Valence		Personal relevance	
	*F* (1, 693)	*p-*value	*F* (1, 694)	*p*	*F* (1, 694)	*p*
**Main effects**						
Mask type	279.87	0.000	460.00	0.000	16.96	0.000
Happened to me	0.07	0.797	0.94	0.332	3.21	0.074
Witnessed it	0.37	0.544	0.77	0.380	3.96	0.047
Learned about it	0.11	0.738	0.30	0.582	0.00	0.996
Part of my job	0.18	0.673	0.04	0.837	0.07	0.793
**Interactions with mask type**						
X Happened to me	0.43	0.514	0.21	0.650	3.48	0.063
X Witnessed it	0.25	0.616	2.60	0.107	4.25	0.040
X Learned about it	0.66	0.418	0.10	0.756	0.13	0.715
X Part of my job	1.04	0.308	0.18	0.674	3.07	0.080

**Table 3:** Results from analysis of covariance examining differences in arousal, valence, and personal relevance ratings by mask type, traumatic event cluster, and the interactions between mask type and trauma event cluster.

	Arousal		Valence		Personal relevance	
	*F* (1, 684)	*p-*value	*F* (1, 694)	*p*	*F* (1, 694)	*p*
**Main effects**						
Mask type	309.30	0.000	441.15	0.000	2.29	0.131
Physical assault	0.01	0.940	0.19	0.660	2.15	0.143
Accident/injury	0.16	0.690	0.14	0.709	0.01	0.944
Natural disaster/sudden death	0.28	0.597	0.61	0.437	0.39	0.533
Sexual violence	0.002	0.964	2.93	0.087	1.94	0.164
Criminal assault	0.03	0.855	2.35	0.126	1.37	0.242
Combat/toxic substance	2.92	0.088	0.03	0.868	2.20	0.138
**Interactions with mask type**						
X Physical assault	0.26	0.607	0.15	0.697	0.08	0.780
X Accident/injury	4.74	0.030	0.43	0.514	0.06	0.807
X Natural disaster/sudden death	0.87	0.352	5.75	0.017	0.004	0.949
X Sexual violence	1.00	0.317	0.65	0.419	2.72	0.099
X Criminal assault	0.76	0.383	0.98	0.323	10.71	0.001
X Combat/toxic substance	1.83	0.177	4.80	0.029	1.68	0.196

## 5 Discussion and implications

This study examined the associations of the types of mask images (depicting SM injury/trauma vs. neutral imagery) on ratings of valence, arousal, and personal relevance. The study also examined associations between responses to images and a personal history of experiencing adverse and traumatic events directly or vicariously (as measured using the LEC-5). The findings revealed that SM-created masks (which included combat, brain injury and PTSD-related imagery) elicited more negative valence and higher arousal compared to neutral masks (which did not have any explicit combat, brain injury, or PTSD-related imagery). This result indicates that the content of the imagery is associated with a significant role in influencing the arousal, valence and personal relevance to viewers. The effect sizes demonstrate that the impact of the mask type on valence and arousal is large. These findings build on work by evidence of differential responses based on viewer’s diagnosis on PTSD (Mueller-Pfeiffer et al., 2013). We demonstrate in this study that even artistic images depicting human-like face in masks can evoke differential responses in terms of arousal and valence. This finding also relates to research by Ardizzi et al. (2021) who reported greater empathic responses to imagery depicting pain which potentially is greater for those who have known the kind of distress being depicted in the images. Humans responds reflexively to what we see (Gallese, 2001, 2003; Freedberg and Gallese, 2007) which in this case were face-like mask images that signal a personal story or mental state can be relatable to the viewer based on their own history of adversity and trauma. The distressing images of fear and anger have been known to resonate more robustly with amygdala activation, a neural center vital for processing emotion (Adolphs, 2008; Osaka et al., 2012). This result would be salient for individuals who have experienced PTSD and thus have a differential response to fear-inducing stimuli (Shin et al., 2006).

An additional finding of significance was that participants who had witnessed trauma perceived the SM created masks to be as personally relevant as the neutral masks, whereas those who had not witnessed a traumatic event found the neutral images to be more personally relevant. These findings suggest that artistic imagery representing traumatic narratives can have a greater emotional impact on individuals who have been exposed to traumatic events. However, it is important to note that our findings did not allow us to conclude whether the personal relevance of trauma mask imagery was stronger for individuals with a personal history of trauma and adversity. Furthermore, the effect size of mask type on personal relevance was small, and the effect size of the interaction between mask type and witnessing trauma was smaller, indicating that the impact of mask type differed only slightly depending on whether the participants had witnessed a trauma. These findings have implications for art therapy and neuroscience research and highlight the importance of being sensitive to and the need to continue to understand the impact of evocative imagery on anyone interacting with individuals who have had experiences of adversity and trauma.

It is of note that about a third of the participants did not complete the survey and it is unclear why this might have occurred. It could have been fatigue or the questions themselves might have been emotionally demanding. The findings also point to the potential of re-traumatization and vicarious traumatization in viewing imagery depicting physical, psychological and moral injuries. While research on the impact of vicarious traumatization on therapists has been discussed for nearly 30 years since the term was coined (McCann and Pearlman, 1990; Pearlman and Mac Ian, 1995), the phenomenon of vicarious traumatization through artwork remains understudied. Drapeau et al. (2022) investigated a response art workshop for psychotherapists who work with survivors of trauma and found that participants identified that learning about vicarious trauma, using response art to engage in self-reflection and self-care, and learning more about themselves and other participants were beneficial outcomes post-workshop. Exposure to imagery representing traumatic stories demonstrated visceral reactions to the imagery as a response to being exposed to their clients’ trauma (Breuche, 2012) and some clinicians art therapists have created response art to express and process their experiences of bearing witness to traumatic stories (Gibson, 2018) and compassion fatigue (Hyatt, 2019).

This study provides insight into the personal relevance of artworks conveying traumatic narratives, particularly for individuals who have witnessed trauma. Healthcare professionals working with communities whose members that have experienced traumatic events may be continually exposed to the lived experiences of their clients while engaging with their artwork. Consequently, viewing artwork that depicts traumatic narratives may have a more profound personal impact on therapists compared to individuals who are not directly involved in witnessing trauma stories. Moreover, the evocative nature of artwork, including artistic masks created by SMs, may lead viewers to experience discomfort, arousal, or vicarious distress. Previous studies in art therapy have found an association between increased empathy and engagement with personally evocative artistic works. Bradshaw (2016) found that art-making processes allowed middle-school students to explore interpersonal and communal issues, thereby fostering the development of empathy. Betts et al. (2015) suggested that the act of viewing one’s own previous artwork can sustain increased levels of empathy. A study focused on artmaking following a visit to the Holocaust Memorial Museum concluded that engaging in artistic activities can serve as a reflective practice, leading to heightened empathy. Even when viewed 1 year after its creation, the artwork could still evoke emotional reactions in viewers (Betts et al., 2015). These taken together indicate that potential of enhanced empathy might co-exist with increased distress especially when individuals find increased personal relevance with works they view. The link and increased potential for various traumatization is potential consideration.

### 5.1 Limitations

This survey study focused on trauma imagery created by individuals who served in the United States military and experienced combat and comparative neutral imagery created by art therapists and arts in health professionals. Individuals who had experienced war, armed conflict, or physical violence may have found the images relatable as seen in higher ratings of personal relevance for these participants. The Life Events Checklist (LEC) used in this study included both non-interpersonal and inter-personal traumas, but these were not differentiated in the analysis. The survey may not have resonated with others, including those with non-combat-related trauma histories. This survey does not encompass all types of adversity and traumatic experiences and may not be representative of diverse populations. Additionally, we did not collect any demographic data so there is limited understanding of how these factors might have affected outcomes. Future research that includes demographic data could provided valuable insights into the characteristics of the sample and helped generalize the findings to larger populations as well as understand potential interactions among factors such as age, gender, ethnicity, cultural background, military service status, and responses to the mask imagery.

Regarding participant attrition, the survey completion rate declined from 1,101 participants responding to the mask questions in the first half of the survey to 699 participants progressing through the LEC at the end. Although approximately 36.5% of the participants dropped out before completing the survey, there were no significant differences in overall valence and arousal ratings between those who completed the full survey and those who only completed the first half. This result suggests that the dropouts may have had reasons other than emotional exhaustion from the images for not completing the survey. Moreover, anecdotal feedback received from some participants who did not complete the survey indicated that the length of the survey itself was one of the reasons why people had difficulty completing it. However, participants who completed the full survey rated the masks as less personally relevant compared to those who did not. It is possible that individuals who found the imagery relevant to their life experiences may have felt the need to discontinue the survey, suggesting vicarious experiences while viewing mask imagery. Overall ratings indicated that participants found the whole sample of masks to be slightly more unpleasant than pleasant, moderately arousing, and relatively low in personal relevance.

In terms of time taken to complete the survey, we found that even though participants were instructed to observe each mask image for a brief 5-s period, many participants exceeded this timeframe, resulting in an average survey completion time of approximately 60 min. Although maintaining a 5-s viewing time was important to capture participants’ initial emotional responses, we had limited control over the actual duration of image viewing. The extended viewing time could also have influenced the attrition rate.

### 5.2 Suggestions for future research

The findings from this study provide evidence that artistic content influences the level of emotional response and that personal experiences of trauma further impact the extent of this response. The findings were in line with research on responses to performance-based assessments such as Rorschach. Hwang et al. (2023) showed that first responders who are at high risk for PTSD due to the nature of their work, are more likely to experience intrusive images and thoughts. Additional research could be conducted on the mechanistic neurological differences between engaging with masks versus human faces that could potentially also examine the role of cultural associations with masks and the way in which they are perceived. Future research can delve into investigating whether the traumatic experience itself or the degree of personal connection to the trauma narratives influence the perception of personal relevance. The inclusion of personal history and demographic information could further shed light on these preliminary findings. Overall, masks with trauma imagery were found to be emotionally evocative in comparison to neutral masks and may serve as a potentially useful tool for emotional evocation in studies of emotional responsiveness. Studies could examine the similarity in viewer responses to faces versus masks including if the FFA (Kanwisher et al., 1997) is activated in similar ways. Viewer responses and activation of the triadic model of esthetic engagement (Chatterjee and Vartanian, 2014) could also be examined. For example, when viewing evocative imagery, we can examine whether and to what extent sensori-motor and meaning making systems are also activated Further study on the neural correlates of sadness, fear, or disgust-related artistic content in masks will be necessary to expound on the neurological processing of these emotions and expressions represented in the masks (Osaka, 2022). It would be worthwhile to consider examining default mode network activity to examine neural responses to esthetic experiences of these masks that concern or match an individual’s sense of identity and ‘self-relevance’ (Vessel et al., 2013).

Future research could also focus on investigating the relationship between art viewing and experiences of different clusters of post-traumatic events and viewer characteristics. In a network analysis of different types of trauma using the LEC, Contractor et al. (2020) found that victimization trauma, including physical and sexual assault by another person, was positively correlated with the severity of PTSD and symptoms of depression, as well as emotional dysregulation. Nuanced exploration of post-traumatic event types may promote comparisons across clinically relevant variables related to PTSD severity, depression, and negative mood dysregulation which may be relevant for developing individualized art therapy interventions. Unlike civilian encounters with trauma, military trauma (for example, deployment to the front lines) may be coupled with other co-morbidities (TBI, etc.). Not knowing health histories of the participants is a limitation as well. For example, both traumatic brain injury (TBI) and post-traumatic stress disorder (PTSD) create profound effects on the structure and function of the brain, influencing how individuals process and respond to visual stimuli. TBI, often resulting from sudden impacts to the head, can lead to defects in primary vision, eye movement, saccadic and smooth pursuit movements, motion vision, and visuo-spatial function (Armstrong, 2018). The visual reaction time of patients with head injuries is slower than that of healthy control individuals (Levin, 1998). White matter integrity is found to be reduced across the spectrum of TBI severity (Kraus et al., 2007) and the integrity of white matter is shown as correlated with visual memory (Spitz et al., 2013).

The findings also underscore the importance of mental health care providers (including art therapists and arts in health professionals) engaging in their own self-care strategies (Hyatt, 2019), because providers with histories of traumatic experiences may become more emotionally activated by artwork depicting traumatic experiences. For example, Hyatt (2019) explored a process of engaging in a life review using response art to address her own life experiences that were activated by the vicarious trauma work she experiences as an art therapist.

This exploratory study also highlights the need to attend to the range of responses to imagery and the particular impact of personal history on emotional activation to visual artwork. It is crucial to consider that people participating in group therapeutic settings likely have varying levels of traumatic experiences. Because people with reported trauma histories were more likely to report increased emotional activation when viewing art depicting another’s traumatic experiences, as demonstrated by this study, group members may have different levels of emotional activation when viewing artwork created by other members of the group depending on their own level of traumatic exposure. Further research may explore levels of emotional activation when viewing art depicting the traumatic experiences of another, and how the varying levels of emotional activation experienced among group members affect their prognosis and wellbeing. In addition, future studies can determine if these differences vary by demographic variables such as age, gender, race/ethnicity, education level, and socio-economic background. The findings have implications for art therapy and arts in health practices, particularly in the use of art viewing as a therapeutic tool. Understanding the nuances of how personal history of trauma influences response to art can lead to more personalized art therapy interventions, as well as open new discussion on displaying client art in public spaces.

Lastly, the visual responses to artwork are clearly influenced by a range of factors including personal experience as well as our innate responsiveness to facial structures and visual symbols (Jacobsen et al., 2006). In this study we used masks, which are reminiscent of the face but also not the face itself (Van den Stock et al., 2008; Zhao et al., 2019) leading to potential interpretations and associations not fully related to the facial recognition area (fusiform gyrus) of the brain. Further research might examine how and to what extent images qualify as traumatic and/or neutral based on visual elements. A detailed analysis of visual elements in the masks that contribute to whether they are perceived as distressing, neutral or positive is a area of further inquiry. This study did not analyze in-depth the specific visual elements that are perceived as traumatizing or neutral and as such that is worthwhile to explore further to better understand what image elements tend to be associated with different emotional responses. In this study we used the SAM to assess emotional responses and associations with the imagery. Future studies might combine open-ended narrative responses along with the rating scales to better understand the participants responses to the images. In addition, this study also calls for further investigation into what images might be in the public domain in civilian and military settings and in healthcare contexts. Images that resonate with human beings who have faced adversity can vary and some images might be both disturbing and comforting depending on the viewers’ life histories. Examining these additional dimensions of imagery in healthcare is also an area of further inquiry.

## 6 Conclusion

This initial study provided insights into the relationship between viewing artistic masks elicited emotional responses elicited. This research offers insights into the relationship between personal trauma history on emotional responses toward art, and the underlying neurobiological mechanisms. By doing so, it not only advances academic knowledge but also has important practical implications for improving therapeutic practices and supporting trauma recovery. The findings indicate that traumatic life events impact the perception of trauma-related imagery, and that neutral imagery overall evokes less of an emotional response in viewers than does imagery depicting injuries. To the best of our knowledge, this study is one of the first to examine the differences in responses to traumatic versus neutral imagery and how the responses vary by the viewers’ own personal history of exposure to adversity and trauma. The findings are of note to healthcare providers and caregivers highlighting vulnerabilities and sensitivities of visual imagery including for individuals with a history of experiencing and/ or witnessing trauma.

## Data availability statement

Data can be made available on request to the corresponding author.

## Ethics statement

The studies involving humans were approved by Drexel University Institutional Review Board. The studies were conducted in accordance with the local legislation and institutional requirements. The participants provided their written informed consent to participate in this study.

## Author contributions

AA: Conceptualization, Data curation, Investigation, Methodology, Project administration, Writing – original draft, Writing – review & editing. BM: Writing – review & editing, Investigation, Methodology, Project administration, Writing – original draft. KS: Writing – review & editing, Data curation, Writing – original draft. JH: Writing – review & editing, Formal analysis, Statistical analysis. HS: Writing – review & editing, Conceptualization. DL: Writing – review & editing. CL: Writing – review & editing. JW: Writing – review & editing. GK: Conceptualization, Data curation, Formal analysis, Investigation, Project administration, Resources, Supervision, Validation, Writing – original draft, Writing – review & editing, Methodology.

## Author disclaimer

The opinions contained herein represent the private views of the authors and are not to be construed as official or as reflecting the views, opinions, or policies of the Henry M. Jackson Foundation for the Advancement of Military Medicine, Inc., the Department of Defense, the National Endowment for the Arts, or the U.S. Government. Mention of trade names, commercial products, or organizations does not imply endorsement by the U.S. Government. This material was created free of branding or market affiliations. The authors are operating solely as contributors. The work of John Williamson was authored as part of their official duties as an employee of the United States Government and is therefore a work of the United States Government. In accordance with 17 USC. 105, no copyright protection is available for such works under US Law.
